# Treatment-Resistant Depression With Catatonia Presenting as a Feature of Creutzfeldt-Jakob’s Disease

**DOI:** 10.7759/cureus.8300

**Published:** 2020-05-26

**Authors:** Muhammad Uneib, Randolph Devereaux, Ian H Rutkofsky, Sourabh Lahoti, Marvin Lopez

**Affiliations:** 1 Internal Medicine, Coliseum Medical Centers, Macon, USA; 2 Research, Coliseum Medical Centers, Macon, USA; 3 Psychiatry, Aventura Hospital and Medical Center, Aventura, USA; 4 Neurology, Coliseum Medical Centers, Macon, USA

**Keywords:** catatonia, creutzfeldt–jakob disease, pseudo-dementia, real-time quaking inverse conversion, 14-3-3, electroconvulsive therapy

## Abstract

A high-functioning middle-aged successful businessman developed a rapid decline in his cognitive, behavioral, and motor abilities within one year. He was initially diagnosed with dementia by a neurologist and was then diagnosed with pseudo-dementia secondary to major depressive disorder with catatonia by a psychiatrist who initiated treatment with Ativan. He was referred to our psychiatric facility for an evaluation to undergo electroconvulsive therapy (ECT) as a potential treatment for medically refractory depression complicated with catatonia and pseudo-dementia. The neurology team and internal medicine team were consulted by a psychiatrist for clearance to begin a course of ECT. In this process, with a coordinated effort and prompt workup and evaluation, including neurological testing, imaging, and positive cerebrospinal fluid analysis for real-time quaking inverse conversion (RT-QuIC) and 14-3-3, the patient was diagnosed with Creutzfeldt-Jakob’s disease.

There are many organic causes of dementia and catatonia that should be explored in depth, especially when the clinical picture is challenging and atypical.

## Introduction

Catatonia was first astutely described by German physician Dr. Karl Ludwig Kahlbaum in 1874 as "tension insanity" [1]. In many Diagnostic and Statistical Manual of Mental Disorders (DSM) versions until 1994, catatonia was described in the context of psychiatric illness mostly associated with schizophrenia [2]. It was not until after 1994 when catatonia secondary to a medical condition was recognized. In DSM V, catatonia is defined as three or more symptoms of catalepsy, waxy flexibility, stupor, agitation, mutism, negativism, posturing, mannerism, stereotypies, grimacing, echolalia, and echopraxia [3]. However, one of the arguments against this method of diagnosis is that the physician will overlook the organic cause of catatonia, and, for this purpose, DSM V also includes catatonia due to a medical condition and catatonia NOS (not otherwise specified) [4].

Recommended treatments of catatonia due to psychiatric conditions continue to be trial of benzodiazepines and electroconvulsive therapy [4]. However, more and more physicians and psychiatrists are identifying catatonia as a sequel of many other medical conditions where benzodiazepines and ECT are not recommended and, in many instances, can make symptoms much worse. A complete workup of organic causes is essential before diagnosing catatonia due to psychiatric illness. One such patient happens to be our patient.

## Case presentation

A middle-aged successful businessman was referred to our hospital for electroconvulsive therapy for treatment-resistant depression. He had a rapid decline in his cognitive, behavioral, and motor abilities 15 months before admission to our hospital. Initially, his wife noticed that he was having unusually high anxiety and developed paranoia. Around the same time, he had a major altercation with his son and he expelled his son from his company. Change in his behavior was attributed to this altercation. His family noticed that he used to forget small things. Four months into his condition, he lost his way to his office a few times and was involved in minor motor vehicle collisions, which was very unusual for a safe driver like him. A month later, he completely lost his way to home and could not get back by himself. His family prohibited him from driving. Later in the same month, he was noted to wander in his neighborhood aimlessly and, on one occasion, with a butcher’s knife in his hand. He was taken to a neurologist. He did not fully participate in the cognitive examination, but the possibility of rapidly progressing dementia was raised. MRI of the head was performed, which was reported to show generalized atrophy and non-specific white matter hyperintensities. MRI spectroscopy was performed, which was reported to show hypometabolism in the bilateral parietotemporal lobes, most commonly seen in Alzheimer’s disease. Investigations for common reversible causes of dementia, such as vitamin B12 deficiency, thyroid dysfunction, heavy metal poisoning, electrolyte derangements, and syphilis, were unremarkable. He was diagnosed to have Alzheimer’s disease by the neurologist, but he did not follow up with him to begin treatment. His altercations with the son continued and his family attributed this decline in his memory and behavior to stress caused by these altercations. He did admit to a close friend that he has been feeling sad and very low for a long time. He expressed his wish to be baptized and sought redemption from the church. He had never been a religious person. He developed short-stepped, slow gait at month 8, since the onset of his condition, which by month 9 progressed to a point where he required assistance to walk. His daughter had a video recording of him getting baptized showing classic Parkinsonian gait. Convinced that his symptoms are due to stress from confrontations with his son, his family took him to a psychiatrist who diagnosed him to have a major depressive disorder with catatonia. He was admitted to inpatient psychiatry unit and intramascular lorazepam was started. He was hospitalized for about three weeks, and there was some improvement in his symptoms according to his wife. At the 12-month mark, he had another major argument with his son, and a couple of days since then he had been mute and bed-ridden with minimal voluntary movement. He was taken to his psychiatrist again who referred him to our hospital for ECT for medically refractory depression and catatonia.

On our initial examination, the patient was disheveled, emaciated, awake but inattentive. He avoided eye contact and did not follow commands. He exhibited monosyllabic echolalia, which was irrelevant to the questioning. He was noted to have full range of eye movements but a hypomimic face with pooling of oral secretions in his mouth. There was appendicular and truncal rigidity with elbows, wrists, and fingers flexed, and knees and ankles extended. These muscles could not be relaxed even with passive movement. Deep tendon reflexes were brisk, whereas plantar reflex was flexor bilaterally. Systemic examination was unremarkable.

**Figure 1:**
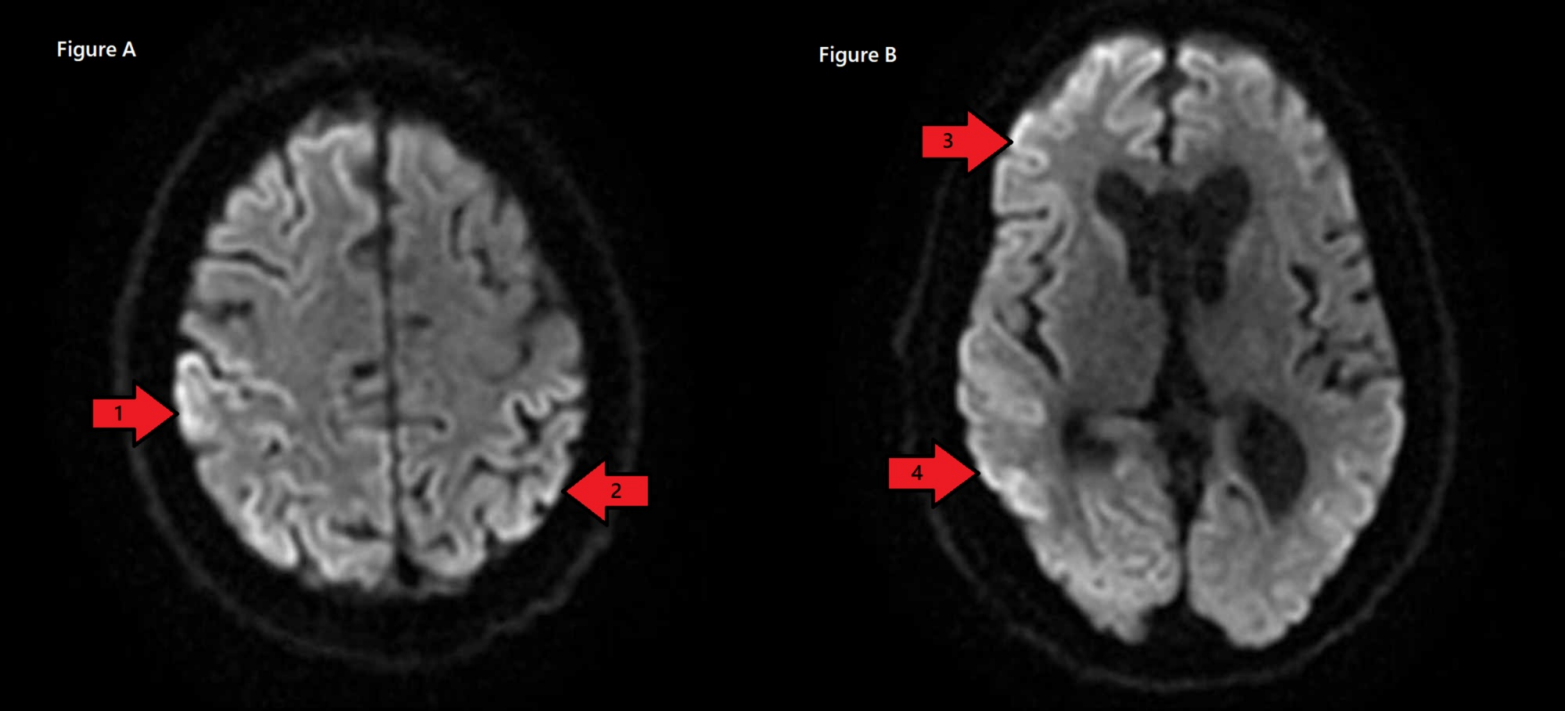
Diffuse T2 hyperintensity on diffusion imaging was observed involving the parietal (A, arrow 1), occipital (A, arrow 2), frontal (B, arrow 3), and temporal (B, arrow 4) cortices.

Lumbar puncture was performed, which was negative for the infectious process. MRI of the head was performed, which showed diffuse T2 hyperintensity on diffusion imaging involving the bilateral frontal, parietal, temporal, and occipital cortices, as seen in Figure 1. No other sequences including FLAIR (fluid-attenuated inversion recovery) showed these changes. Radiologist raised the possibility of an artifact, and MRI was repeated. Repeat MRI was unchanged. All other metabolic workup was negative, including normal sodium, negative urine analysis, and negative HIV.

The patient was empirically treated with Ativan 2 mg three times a day for catatonia without resolution of his symptoms. Up to this point, the family understood that the patient had catatonia from severe depression and also that there was undue guilt with his son, daughter, and wife that the patient had gotten worsening of symptoms after arguing with them on different occasions. Every day the family would show us pictures and videos of once healthy, proud, loving husband and father. At this point, the possibility of Creutzfeldt-Jakob’s disease (CJD) was raised. Our patient had rapidly progressing dementia, cortical ribboning on the MRI, and cerebrospinal fluid (CSF) analysis positive 14-3-3. The patient’s CSF was sent for real-time quaking inverse conversion analysis (RT-QuIC) (more on this in the Discussion section). While awaiting results, Ativan was continued without change in the patient's catatonic state. RT-QuIC was positive, and based on the criteria for CJD by the Centers for Disease Control and Prevention (CDC), a diagnosis of CJD was made.

## Discussion

**Figure 2:**
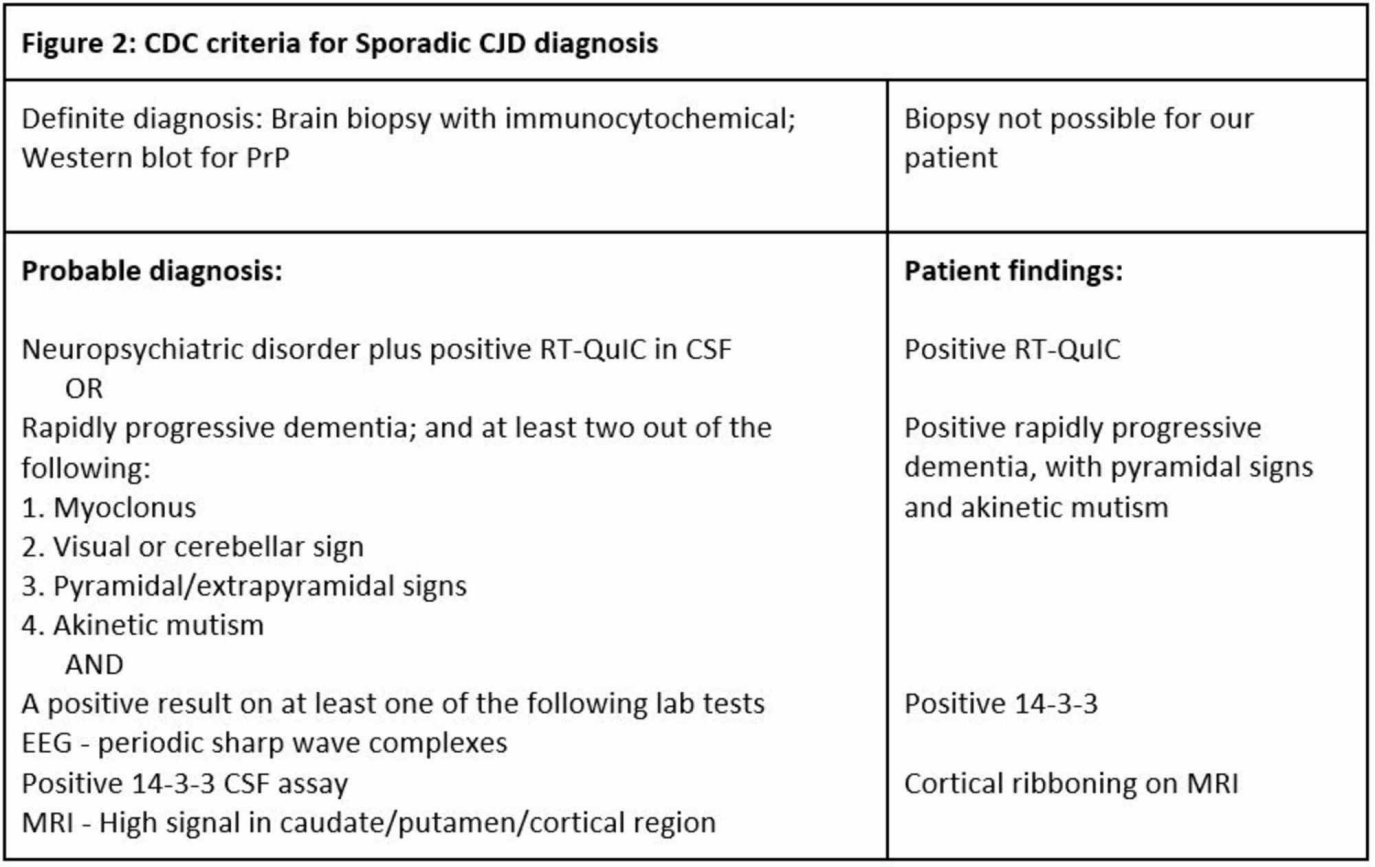
CDC criteria for Sporadic CJD diagnosis. CDC, Centers for Disease Control and Prevention; CJD, Creutzfeldt-Jakob’s disease; RT-QuIC, real-time quaking inverse conversion; CSF, cerebrospinal fluid

A highly-functional middle-aged man was referred to our hospital with a diagnosis of major depressive disorder with catatonia but was discovered to have CDJ. CDJ is a rare neurodegenerative fatal disorder that approximately affects about 350 patients every year in the United States [5]. The most common form of CJD is sporadic CJD, which comprises 85% of the cases, and within the year of illness most of these patients die [6]. CJD is the condition of protein (PrP) misfolding, and PrP is more abundantly found in the brain tissue. In early stages, 30% of patients exhibit personality issues that can be confused with psychiatric illness, and in late stages, up 57% can be confused with psychiatric illness [7]. Clinical diagnosis of CJD can be made by the criteria outlined by the CDC, and these criteria can be found in Figure 2 [5]. Our patient fulfilled the probable criteria listed in Figure 2. Brain biopsy would have provided a definitive diagnosis; however, it was not an option.

Our patient tested positive for RT-QuIC. RT-QuIC has emerged as the most accurate laboratory test for the diagnosis of CJD. The RT-QuIC assay has a diagnostic sensitivity of 96% and a specificity approaching 100% [8]. The assay is a 96-well plate, with multiple products that promote the formation of PrP in the well if the patient's CSF has PrP. Once the well plate is seeded with the patient's CSF, it is placed in the mixing machine that further promotes the PrP formation. The well plate also has an amyloid sensitive dye, which fluoresces once PrP aggregates in large quantities [8].

From the perspective of the patient’s family, RT-QuIC helped in the diagnosis, and they were counseled regarding the patient's poor prognosis and lack of treatment. The family was grateful that instead of guilt they could get closure as a family. Now they could celebrate his amazing life with him in his last moments. His family took him home with hospice support.

## Conclusions

Severe depression with catatonia can be a presenting feature of CJD. It is of vital importance to rule out organic causes of catatonia before attributing it to primary psychiatric illness. CJD is a rare but important cause of akinetic mutism and it should be considered in all the patients presenting as such. It can be diagnosed with the help of a well-defined clinical criterion and highly specific laboratory and radiological tests. Appropriate and timely diagnosis can prevent numerous unwarranted tests and treatments, which can further increase the suffering of the patient.
